# Chronic Thromboembolic Pulmonary Disease: Right Ventricular Function and Pulmonary Hemodynamics in a 4-Year Follow-Up

**DOI:** 10.3390/ijms262110617

**Published:** 2025-10-31

**Authors:** Rosalinda Madonna, Giorgia Tocci, Filippo Biondi, Viola Cipollini, Riccardo Morganti, Raffaele De Caterina

**Affiliations:** 1 University Cardiology Division, Pisa University Hospital and University of Pisa, Via Paradisa, 2, 56124 Pisa, Italy; g.tocci3@studenti.unipi.it (G.T.); f.biondi9@studenti.unipi.it (F.B.); v.cipollini@studenti.unipi.it (V.C.); 2 Statistics Unit, Pisa University Hospital, 56100 Pisa, Italy; r.morganti@ao-pisa.toscana.it

**Keywords:** chronic thromboembolic pulmonary disease, Q-scan, right ventricular dysfunction, exercise-induced pulmonary hypertension, cardiopulmonary exercise test, exercise echocardiography

## Abstract

Chronic thromboembolic pulmonary disease (CTEPD) with or without pulmonary hypertension (PH) is characterized by persistent perfusion defects and progressive pulmonary vascular dysfunction after acute pulmonary embolism (PE), despite adequate anticoagulant therapy. We aimed at assessing clinical, hemodynamic, and functional evolution in patients screened for CTEPD with persistent lung perfusion scintigraphy (Q-scan) defects to identify non-invasive predictors of right ventricular (RV) impairment and development of exercise-induced pulmonary hypertension (ExPH). We analyzed 55 patients with a history of PE and no prior cardiopulmonary disease, stratified by perfusion (Q)-scan at 4 months into Q-scan-positive (n = 35) and Q-scan-negative (n = 20). At that time, all patients underwent echocardiography, cardiopulmonary exercise testing (CPET), and exercise stress echocardiography (ESE). Clinical evaluation and resting echocardiography were repeated at 24, 36, and 48 months. At baseline, Q-positive patients had higher NT-proBNP levels and greater PESI scores. At 4 months, they exhibited a higher prevalence of exercise-induced pulmonary hypertension (ExPH) on both CPET and ESE (*p* < 0.001). Both groups showed a partial recovery of echocardiographic parameters over time; however, Q-positive patients featured significantly higher systolic (s) pulmonary artery pressure (PAP) and mean PAP and a lower TAPSE/sPAP ratio (*p* < 0.001), increased eccentricity index, and shorter right ventricle (RV) outflow tract acceleration time at 48 months, suggestive of persistent RV-PA uncoupling and of a higher subclinical hemodynamic burden. Persistent Q-scan defects identify a post-PE population at risk for long-term RV dysfunction and ExPH, even in the absence of pulmonary hypertension at rest. CPET and ESE at 4 months provide useful prognostic information, supporting their integration into structured follow-up strategies to identify patients early on with evolving pulmonary vascular disease.

## 1. Introduction

Long-term pulmonary complications of pulmonary embolism (PE) are collectively referred to as post-pulmonary embolism syndrome (PPES). This embraces two distinct, mutually exclusive clinical entities: post-PE-related dyspnea and chronic thromboembolic pulmonary disease (CTEPD), which differ for the presence of thrombotic obstructions after at least 3 months of effective, post-PE anticoagulation [1]. Depending on the value of mean pulmonary artery pressure (mPAP), CTEPD is, in turn, classified as CTEPD without hypertension (CTEPD wo PH) or chronic thromboembolic pulmonary hypertension (CTEPH), the main form of group 4 pulmonary hypertension (PH).

Current guidelines recommend that PE patients who remain symptomatic at completion of 3 months of effective anticoagulation or carry risk factors for CTEPH be screened for residual thrombotic obstructions of the pulmonary vessels by ventilation/perfusion (V/Q) scintigraphy and transthoracic echocardiography (TTE). Patients with thrombotic obstructions at V/Q scan and an intermediate or high probability of PH at TTE should proceed to right heart catheterization (RHC) so as to establish a diagnosis of CTEPD wo PH, if mPAP < 20 mmhg, or CTEPH, if mPAP exceeds this threshold [1].

If, on the one hand, a great effort has been made to characterize and treat CTEPH [2], the clinical management of post-PE-related dyspnea and CTEPD wo PH is beset by a shortage of evidence regarding their epidemiology, natural history, clinical characteristics, hemodynamic correlates, and treatment. A retrospective, registry-based study by Nilsson et al. showed that 68% of PE patients remained symptomatic at a median time from diagnosis of 3.4 years [3]. Dzikowska-Diduch et al. [4,5] authored a key, observational longitudinal study on PPES, which followed a PE cohort for 6 months. More than half of PE patients remained symptomatic at 6 months. CTEPH and CTEPD wo PH were diagnosed in 8.4% and 3.3% of PPES patients who completed the diagnostic work-up, respectively.

The pathophysiological background of long-term dyspnea in PE patients has been elusive [6]. Puzzlingly, PE severity does not seem to have a significant association with the degree of long-term functional limitations [7]. Even less clear is the reason behind the persistence of thrombotic occlusions in some symptomatic patients under the same post-PE guideline-directed anticoagulation regimen. A prospective observational study by our group on a PPES cohort [8] showed that patients with persistent lung perfusion scintigraphy (Q-scan) defects had higher PESI scores at admission during a PE index event, while at the 24-month follow-up, they had higher levels of NT-proBNP, a worse functional class, worse right ventricle–arterial coupling, and diastolic dysfunction compared to post-PE-dyspnea. Furthermore, stress echocardiography (ESE) and cardiopulmonary exercise testing (CPET) were able to effectively distinguish between patients with positive screening for CTEPD and post-PE dyspnea, with nearly all of the former showing an ESE and CPET pattern compatible with exercise-PH [8]. It has to be noted, however, that the prevalence of exercise PH in patients with CTEPD wo PH assessed through exercise RHC, which remains the only modality that can lead to a diagnosis ex-PH [1], did not exceed 21% in a recent study on a small cohort of PPES patients [9].

The present study is the long-term continuation of our earlier, proof-of-concept, observational study on a small PPES cohort [8]. We set out to compare baseline and follow-up characteristics of symptomatic post-PE patients who had a positive screening for CTEPD with those of patients with post-PE dyspnea who did not show persistent thrombotic obstructions at perfusion scintigraphy. We aimed to investigate clinical and echocardiographic parameters, which may help predict the evolution of PE into its possible long-term complications. Specifically, we aimed to (1) identify baseline characteristics that may account for and help predict the persistence of thrombotic defects in post-PE cohorts; (2) track the short and long-term impact of residual perfusion defects on pulmonary hemodynamics and prognostically-relevant clinical and laboratoristic parameters; (3) investigate whether patients with thrombotic defects present with a more impaired hemodynamic and functional profile as assessed by noninvasive exercise testing, i.e. ESE and CPET. The overarching hypothesis, is that CTEPD and post-PE dyspnea may follow a divergent natural history, with the former evolving towards a more impaired hemodynamic and clinical phenotype. Moreover, a detailed and multimodal longitudinal follow-up including non-invasive exercise testing can help uncover differences in the pathophysiological background of CTEPD vs. post-PE dyspnea.

## 2. Results

### 2.1. Patient Recruitment and Population Characteristics

Fifty-five patients were included in this study, and all of them completed a 48-month follow-up. CPET was not performed or could not be evaluated in eight patients due to patient refusal, inability to cycle due to concomitant orthopedic conditions, or interruption due to early exhaustion. These patients were not excluded from result analysis. As shown in Table 1, the population was homogeneously selected in terms of sex (*p* = 0.064), age (*p* = 0.053), and BMI (*p* = 0.940). Regarding comorbidities and cardiovascular risk factors, no significant differences were found for smoking status, diabetes, dyslipidemia, hypertension, ischemic heart disease, or valvular diseases. Additionally, the population was homogeneous in terms of cardiovascular drug use (ACE inhibitors, sartans, beta-blockers, diuretics, statins), history of cardiovascular surgery, and other comorbidities such as hematological and oncological disorders, thyroid dysfunction, pulmonary disease. Among PE-related risk factors, the presence of persistent risk conditions and unprovoked embolic events were significantly associated with a positive Q-scan, as was a history of prior venous thromboembolism (deep vein thrombosis or other thrombotic events). A statistically significant difference also emerged in the presence of temporary thrombotic risk factors, which were more prevalent in the Q-positive cohort (11 vs. 1; *p* = 0.022). Q-positive patients were more likely to have suffered from VTE in the past, with the largest differences in the relative frequency of past superficial venous thrombosis combined to deep venous thrombosis (11 vs. 0) and PE (8 vs. 3), with an overall *p* = 0.009 for all VTE events. Use of oral contraceptives, a known transient risk factor for thromboembolism, was also evenly distributed. Of note, the Q-positive group had a non-statistically significant association with a higher age (67.26 (18.68) years vs. 59.40 (10.80) years, *p* = 0.053).

### 2.2. Baseline Assessment

As shown in Table 2, at the time of hospital admission, from a clinical standpoint, patients with a positive Q-scan showed a significantly higher heart rate (*p* < 0.001) and lower systolic and diastolic blood pressure (*p* = 0.062 and *p* = 0.006, respectively); the distribution of WHO functional classes was also markedly different, with a predominance of worse classes in the positive group (*p* < 0.001). Regarding parameters pertaining to PE management and in-hospital stay, Q-positive patients had a more severe disease, as shown by a higher prevalence of massive thrombotic obstruction at CTPA at PE diagnosis (23 vs. 2; *p* < 0.001) and higher PESI scores at admission (*p* < 0.001). Additionally, in the positive group, significantly higher NT-proBNP levels (*p* = 0.003) along with a higher prevalence of heart failure (*p* = 0.006) support the hypothesis of increased hemodynamic overload of the right heart due to an elevated pulmonary pressure, further confirmed by a significantly higher probability of resting pulmonary hypertension in this group (*p* < 0.001). During the acute phase of TEP, the administration of fibrinolytics and heparin was significantly more frequent in the positive group (*p* = 0.001 and 0.003, respectively).

As shown in Table 3, at baseline, patients with a positive lung perfusion scan already showed significant echocardiographic alterations suggestive of early right ventricular (RV) involvement and pulmonary circulatory impairment. Specifically, the positive group exhibited a marked increase in RV diameters and outflow tract (RD1, RD2, RD3, proximal and distal RVOT, with *p*-value < 0.001), and an important RV systolic dysfunction, as evidenced by a significantly reduced tricuspid annular plane systolic excursion (TAPSE: 14.29 ± 2.28 mm vs. 22.10 ± 4.85 mm; *p* < 0.001) and fractional area change (FAC%: 31.34 ± 5.65% vs. 50.70 ± 10.63%; *p* < 0.001), along with a significantly lower TAPSE/sPAP ratio (0.32 ± 0.06 vs. 0.98 ± 0.42; *p* < 0.001), indicating early ventriculo-arterial uncoupling. Signs of increased pulmonary vascular load were also evident, with elevated systolic and mean pulmonary arterial pressures in the positive group (sPAP: 44.74 ± 2.60 mmHg vs. 27.45 ± 10.11 mmHg; *p* < 0.001; mPAP: 29.00 ± 1.64 mmHg vs. 18.65 ± 6.27 mmHg; *p* < 0.001). Regarding RV diastolic function, the E/e’ ratio was significantly higher in the positive group (16.77 ± 1.14 vs. 8.2 ± 1.7; *p* < 0.001), suggesting impaired diastolic filling. In addition, in the context of RV dysfunction, a reduction in forward stroke volume was observed (FwSV: 69.17 ± 7.61 mL vs. 80.95 ± 10.32 mL; *p* = 0.009), likely reflecting decreased left ventricular preload secondary to impaired RV output. Finally, acute-phase computed tomography pulmonary angiography (CTPA) confirmed the perfusion abnormalities later observed on a Q-scan (*p* < 0.001), as shown in Table 1.

### 2.3. Four-Month Follow-Up

#### 2.3.1. Clinical and CPET Parameters at 4 Months

As expected, NT-pro-BNP declined at follow-up, but the inter-group differences not only remained statistically significant, but increased (1256 + −562 ng/mL vs. 94 + −134, *p* > 0.001). Puzzlingly, the WHO functional class improved in both groups, but the gap with the Q-positive group was still significant (*p* = 0.013). Performance at the 6MWT was also worse in the Q-positive cohort (469 m + −146 m vs. 684 m + −52 m; *p* < 0.001) (Table 4).

As detailed in the Methods section, CPET was performed at 4 months (Table 5). The two groups differed with respect to all investigated variables, with the exception of end-tidal carbon dioxide (PET CO_2_), which was at the upper limit of the normal range in both groups (6.2 + −3.4 vs. 6.3 + −8.9) and showed large standard deviations. Peak oxygen consumption (peak VO_2_), the slope of ventilatory equivalents to CO_2_ production ratio (VE/CO_2_ slope), the peak oxygen pulse (peak VO_2_ pulse), the estimate of the ratio of physiologic dead space over tidal volume (VD/VT), and the slope of heart rate to oxygen consumption ratio (HR/VO_2_ slope) were all outside of their reference range in Q-positive patients. Of note, 24/29 Q-positive patients showed a CPET profile compatible with pulmonary vascular disease, except for the PETCO_2_, which was higher than expected in PH.

#### 2.3.2. Resting and Exercise Echocardiography

As shown in Table 6, TTE at rest documented statistically and clinically significant differences between the two groups in terms of right ventricular structure and function. Patients with a positive Q-scan showed an increase in RV diameters and outflow tract (RD1, RD2, RD3, proximal and distal RVOT, with *p*-value < 0.001), a reduced TAPSE (15.29 ± 2.01 mm vs. 22.00 ± 3.26 mm; *p* < 0.001), and a lower FAC% (32.94 ± 5.26 vs. 56.60 ± 10.56; *p* < 0.001). RV diastolic function was also impaired, as shown by a higher E/e’ ratio (17.23 ± 1.24 vs. 8.1 ± 1.83; *p* < 0.001) and a lower E/A ratio (*p* < 0.001). Indirect measures of pulmonary pressure, including sPAP (43.29 ± 2.18 mmHg vs. 24.10 ± 6.24 mmHg; *p* < 0.001) and mPAP (28.00 ± 2.14 mmHg vs. 16.12 ± 4.30 mmHg; *p* < 0.001), and additional parameters for estimating the echocardiographic probability of pulmonary hypertension, such as TR velocity (6.40 ± 1.28 m/s vs. 2.09 ± 0.36 m/s; *p* < 0.001) and RVOT Doppler acceleration time (3.32 ± 0.1 m/s vs. 1.94 ± 0.45 m/s; *p* < 0.001), were significantly higher in the positive Q-scan group than in the negative group. No significant differences were observed in left ventricular size or systolic function. TAPSE/sPAP, a surrogate marker of right ventricle to pulmonary artery coupling [10], was lower in the Q-positive group, yet above the value which was shown to hold prognostic significance in CTEPH [11]. A very high degree of concordance was found between Q-scan positivity and the presence of exercise limitation compatible with ex-PH. Indeed, 24/24 patients with a positive Q scan had an mPAP/CO slope greater than 3 mmHg/min/L at ESE, and 24/28 showed signs of cardiopulmonary impairment at CPET (*p* < 0.001 for both; Table 5 and Table 6).

### 2.4. Long-Term Follow-Up

With regard to NT-proBNP, data are available up to 24 months of follow-up. NT at 24 months was 520.1 ± 261.0 pg/mL in the Q-positive group and 56.8 ± 34.5 pg/mL in the Q-negative group. A significant decrease in levels is observed in both groups between 4 months (T1) and 24 months (T2), with a more pronounced reduction in the positive Q-scan group (from 1256.2 ± 562.5 pg/mL to 520.1 ± 261.0 pg/mL; *p* < 0.001) compared to the negative group (from 94.7 ± 134.4 pg/mL to 56.8 ± 34.5 pg/mL; *p* < 0.001), likely related to the progressive resolution of the acute hemodynamic overload and to adaptive cardiac remodeling mechanisms following the embolic event. The comparison between groups remained significant at 24 months (*p* < 0.001), with persistently elevated and pathological absolute values in the positive group.

Regarding clinical parameters, they improved in both the positive Q-scan and in the negative Q-scan group at 48-month follow-up. However, the WHO functional class (WHO-FC) at 48 months remained significantly different between the two groups, being worse in patients with a positive Q-scan (*p* = 0.029) (Table 7, Table 8 and Table 9).

**Figure 1:**
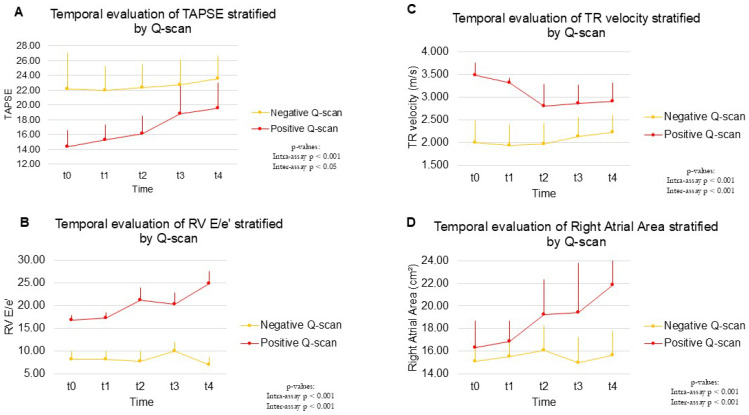
Temporal evaluation of right ventricle echocardiographic parameters stratified by Q-scan. (**A**) Temporal evaluation of TAPSE stratified by Q-scan. Abbreviations: Mean tricuspid annular plane systolic excursion values (±standard deviation) at five timepoints: baseline (t0, acute event), 4 months (t1), 24 months (t2), 36 months (t3), and 48 months (t4) in patients stratified by scintigraphy. TAPSE, tricuspid annular plane systolic excursion. (**B**) Temporal evaluation of RV E/e’ stratified by Q-scan. Abbreviations: Mean RV E/e’ values (±standard deviation) at five timepoints: baseline (t0, acute event), 4 months (t1), 24 months (t2), 36 months (t3), and 48 months (t4) in patients stratified by scintigraphy. RV, right ventricle; E wave, peak filling flow velocity of the ventricle in early diastole (passive filling); e’ wave, early diastolic velocity of the mitral or tricuspid annulus. (**C**) Temporal evaluation of TR velocity stratified by Q-scan. (**D**) Temporal evaluation of Right Atrial Area stratified by Q-scan. Mean TR velocity values (±standard deviation) at five timepoints: baseline (t0, acute event), 4 months (t1), 24 months (t2), 36 months (t3), and 48 months (t4) in patients stratified by scintigraphy. Abbreviations: TR, tricuspid regurgitation.

In the long term, patients with a positive Q-scan showed an improvement in several echocardiographic parameters compared to T1, while still maintaining pathological values significantly higher than in the negative group, which confirm a persistent structural and functional alteration in the positive Q-scan group. In particular, regarding right ventricular remodeling, a reduction in diameters (RD1, RD2, RD3; all *p* < 0.001) and an increase in longitudinal systolic function (TAPSE: +30.46 ± 29.73% in positives vs. +9.03 ± 22.81% in negatives; *p* = 0.007) are observed, with absolute values at 48 months significantly worse in the positive group (e.g., RD3: 32.8 ± 11.5 mm vs. 27.1 ± 4.5 mm, *p* = 0.012; TAPSE: 19.6 ± 3.4 mm vs. 23.5 ± 3.1 mm, *p* < 0.001) (Figure 1; Table 6, Table 7, Table 8 and Table 9).

At the level of pressure overload, a reduction in tricuspid regurgitation velocity is noted (TRV: –12.1 ± 11.9%, *p* < 0.001), along with an increase in right ventricular outflow tract acceleration time (RVOT AT: +29.8 ± 33.3%, *p* < 0.001). Despite this improvement, these values remained pathological in the positive group at 48 months (TRV: 2.92 ± 0.40 m/s vs. 2.23 ± 0.39 m/s; *p* < 0.001; RVOT AT: 85.7 ± 25.5 ms vs. 118.8 ± 9.5 ms; *p* < 0.001), unlike the negative group, which showed complete normalization (Figure 1).

The right-to-left ventricular diameter ratio (VD/VS) and the eccentricity index (EI) confirmed the persistence of overload-induced remodeling in the positive group (*p* = 0.005 and *p* < 0.001, respectively), suggesting chronic septal deformation and relative right ventricular dilation compared to the left ventricle in the positive Q-scan group (Figure 1; Table 7, Table 8 and Table 9).

Furthermore, patients with a positive Q-scan showed a persistently pathological hemodynamic profile, characterized by elevated systolic and mean pulmonary arterial pressures (sPAP and mPAP), and a persistently reduced TAPSE/sPAP ratio, which remained unchanged from baseline to 4-year follow-up, with a *p*-value < 0.001. These parameters indicate an unresolved right ventricular dysfunction and a lack of recovery of the normal relationship between right ventricular function and pulmonary afterload, suggesting RV-PA uncoupling. Right ventricular diastolic function and filling also remained impaired in the positive group. The E/A ratio showed mild improvement in the negative group, in contrast with the reduction observed in positives (*p* = 0.025); however, the statistically significant difference between the two groups persisted at 48 months (*p*-value < 0.001) (Table 9). The E/e’ ratio increased significantly in the positive group (*p* < 0.001), reflecting a persistent elevation in filling pressures (Figure 1). Furthermore, the diameter of the inferior vena cava (IVC) increased significantly (+10.5 ± 22.6% vs. –4.8 ± 9.2%; *p* = 0.001), consistent with an elevated central venous pressure. Finally, a significant increase in right atrial area is observed in the positive group compared to the negative group (*p* < 0.001), in line with chronic remodeling secondary to volume overload (Figure 1).

## 3. Discussion

In this observational longitudinal study, we investigated the long-term evolution of a cohort of patients with a history of acute pulmonary embolism (PE), stratified by the presence or absence of residual perfusion defects at a pulmonary perfusion Q-scan four to six months after discharge. As detailed in the Results section, the two cohorts exhibited a high number of statistically significant differences across the whole scope of tests and at every and each time point. This suggests that CTEPD and post-PE dyspnea stem from a common inciting event (PE), but diverge immediately with regard to clinical, laboratory, and imaging findings.

Concerning baseline characteristics, the higher prevalence of past venous thrombotic disease may indicate that patients who go on to develop thrombotic defects may in fact have suffered from subclinical or undiagnosed pulmonary embolism. This has already been suggested by several studies, showing a higher risk of CTEPH in cohorts affected by recurrent PE [12,13]. Similarly, the association between unprovoked PE and CTEPD has already been described in the past [14] and can be explained by the fact that a lack of a recognized cause of VTE implies a persistent predisposition to clotting. As a result, great attention should be paid before excluding CTEPD after PE in patients with unprovoked VTE and/or a history of recurrent venous thrombosis.

Echocardiography at discharge showed that patients who will go on to exhibit persistent thrombotic defects are more likely to exhibit signs of ongoing RV dysfunction (lower TAPSE and FAC) and ventriculo-pulmonary decoupling (lower TAPSE/sPAP ratio). Our study aligns with results from Kokalj et al. [15], who found that almost half of PE patients show signs of RV dysfunction at predischarge echocardiography. Of note, TAPSE improved in the positive Q-scan group despite deterioration in RV stiffness and progressive RA enlargement. This may indicate that the right ventricle adapts to the increased afterload and reduced preload by augmenting systolic function, such that the observed increase in TAPSE does not represent a true improvement in RV performance, but rather a compensatory phase of enhanced contractility.

Clinical, laboratory, and imaging exams at 4 and 48 months’ follow-up showed a tendency toward improvement in both groups, but patients with positive Q-scan findings exhibited persistent signs of right ventricular dysfunction, with statistically significant differences still evident at 48 months. NT-proBNP levels and the WHO functional class also remained impaired in this group, suggesting a persistent hemodynamic burden.

The strong association between the persistence of thrombotic defects and a worse functional and hemodynamic impairment as assessed by CPET and ESE complements data collected at rest and can inform our understanding of CTEPD pathophysiology.

The use of ESE to look for the signs of ex-PH is not currently recommended by PH guidelines, due to issues including test reproducibility and lack of sufficient clinical validation. However, a number of studies have investigated the application of ESE to PH diagnosis and prognostication. Argiento et al. [16] have proven the technical feasibility of non-invasive pulmonary hemodynamic evaluation at exercise, showing good intra- and interobserver concordance for both sPAP and pulmonary blood flow. Moreover, several recent clinical studies have attempted to validate ESE with respect to clinical endpoints. These include a longitudinal study by the RIGHT-NET group that has showed that an mPAP/CO slope greater than 5 mmHg*min/L effectively predicts a composite of all-cause mortality or HF hospitalization in a composite cohort of patients. Saito et al. [17] have drawn a very similar conclusion in a cohort of HFpEF patients, in which an mPAP/CO slope value of 5.1 mmHg*min/L was shown to predict the same composite endpoint. Kusunose et al. [18] documented the ability to non-invasively assess ExPH, defined as an mPAP/CO slope greater than 3.3 mmHg*min/L, to predict a shorter time to PH development in patients with connective tissue disease. Falter et al. [19] have recently showed the impact of the mPAP/CO slope on survival in a cohort of patients affected by unexplained dyspnea. Evidence specifically supporting the role of non-invasively assessed exPH in post-PE patients is extremely limited. To the best of our knowledge, one study enrolled a cohort of both PAH and CTEPH patients [20] and showed the association of an sPAP increase lower than 30 mmHg with a worse survival. Nonetheless, patients affected by CTEPH were a small percentage of the total cohort, so that the results cannot be assumed to apply to CTEPH. In this context of a general lack of information regarding exercise testing in post-PE patients, our study offers some useful insights. The perfect correspondence between thrombotic obstruction and non-invasively assessed ex-PH suggests that CTEPD go on to develop exercise PH in the early post-PE phase, while patients without persistent thrombi preserve a normal pulmonary hemodynamic at exercise and a more favorable profile at CPET. This finding raises the question whether early post-PE ex-PH persists at a later stage and may be leveraged in post-PE screening for CTEPH. Because of the limitations of the present study, including the lack of patients with thrombotic defects and a normal mPAP/CO slope, this remains a matter of speculation. Notably, the Q-positive cohort had a higher mean age than the Q-negative cohort. Only further studies, enrolling a larger post-PE cohort and conducting ex-RHC, ESE, and CPET exams at different timepoints, may clarify this possibility and offer insights of great clinical value.

### Limitations

This study has several limitations. First, the sample size is modest, which may limit the generalizability of the findings. Second, screening for CTEPD was not conducted in full accordance with current ESC/ERS guidelines on PH, since ventilation scintigraphy was substituted by a chest X-ray, as is common clinical practice in our center. Third, and most importantly, RHC was not performed in the study cohort, which impairs our ability to rule in/out the presence and degree of pulmonary hypertension in Q-scan-positive patients. As a result, the true prevalence of CTEPD with/without PH within this cohort remains unknown. Moreover, the Q-positive cohort had a higher mean age than the Q-negative cohort. This could partly explain the results, especially the findings concerning functional capacity, i.e., WHO FC scores. Because the cohorts had a comparable prevalence of coronary artery disease, it would have been advisable to quantify elderly frailty through dedicated scores and to collect data on non-CV morbidity. These limitations affect the interpretation of the findings and limit definitive conclusions regarding the natural history of post-pulmonary embolism syndrome.

## 4. Materials and Methods

### 4.1. Study Design and Data Collection

This was a prospective, observational, cohort, single-center study that enrolled patients with acute pulmonary embolism (PE) at the Cardiology Division of the University Hospital of Cisanello, Pisa University, in Pisa. This study was conducted in accordance with the Declaration of Helsinki. All procedures were approved by the local Institutional Ethics Committee for Human Studies (Protocol code PH-HF released by CEAVNO).

All patients provided informed consent before each diagnostic test, which was carried out exclusively for clinical purposes. Local investigators had full access to patient data and medical records. Enrollment took place between 2020 and 2024, and 55 patients were included based on the following inclusion criteria: diagnosis of acute pulmonary embolism, eligibility for effective post-PE anticoagulation, and age between 18 and 85 years. Patients with the following criteria were excluded: conditions that prevent the performance of exercise testing, limited life expectancy, and moderate to severe primary tricuspid regurgitation. Patients were selected at different time points among those admitted to the cardiology division of the University Hospital of Cisanello, Pisa University, in Pisa, Italy, with a diagnosis of PE in 2020. At discharge, patients were prescribed anticoagulation therapy in accordance with current guidelines for PE. After 4–6 months, patients who had completed an effective anticoagulation course were screened for symptoms of cardiorespiratory impairment attributable to the index PE event and underwent lung perfusion scintigraphy (Q-scan). Instead of ventilation scintigraphy, a chest X-ray was performed as per routine practice in our Nuclear Medicine Unit. This procedure is permitted in clinical practice by the 2015 ESC guidelines and remains in effect with the 2022 guidelines in centers that cannot rely on V/Q scintigraphy, being supported by solid evidence [10,21]. Patients were classified as Q-scan-negative (group 1, n = 20) or Q-scan-positive (group 2, n = 35). Both groups underwent transthoracic echocardiography (TTE) at rest, and were subclassified based on the probability of pulmonary hypertension (PH) as high, intermediate, or low. Patients with a PH probability (n = 3) were excluded and informed of the need for right heart catheterization (RHC). Those with an intermediate or low PH probability underwent exercise testing, with exercise echocardiography (ESE) performed on the same day as TTE. CPET was carried out 4 months after the acute event. In accordance with current PH guidelines (ESC/ERS 2022), patients with a positive Q-scan, intermediate and high PH probability, and positive CPET were informed about the possibility of undergoing RHC and instructed on the risk–benefit ratio of the test. Group 1 and group 2 were compared based on the following parameters:

1. Extent of thrombotic load of PE on Q-scan (number of segments with perfusion defect) and contrast-enhanced CT pulmonary angiography at admission (mild, sub-massive, or massive PE) according to the American Heart Association definitions [11];
2. Presence of thrombophilia (limited to tests not affected by anticoagulation, namely factor V Leiden, prothrombin variant, anti-phospholipid, and anti-beta-2-glycoprotein antibodies);
3. Cardiovascular risk factors;
4. Anthropometric and demographic parameters;
5. Anticoagulation treatment in the hospital and at discharge.

The two patient groups were also compared for World Health Organization functional class (WHO-FC), NT-proBNP, CPET parameters, and echocardiographic parameters at rest and at exercise (ESE).

**Figure 2:**
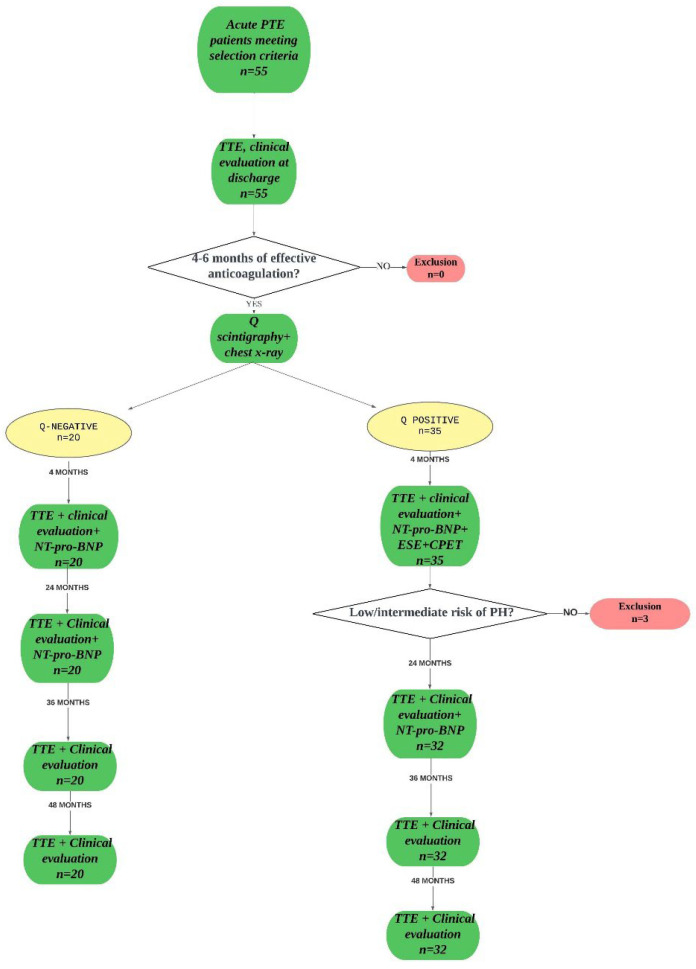
The flow-chart of the study.

Subsequently, enrolled patients were followed up at 24, 36, and 48 months post-admission, during which a resting TTE, clinical examination, and blood tests were repeated. A flow-chart of the study is shown in Figure 2. As shown in the figure, no death or loss to follow up happened for the all duration of the study.

### 4.2. Resting Echocardiography

Mono- and two-dimensional transthoracic echocardiography (TTE) was performed using a Philips iE33 echocardiograph (Philips xMATRIX echocardiography system, Andover, MA, USA). Images were recorded over at least 3 cardiac cycles. The echocardiographic probability of pulmonary hypertension (PH) was assessed according to current guideline criteria [1]. Right atrial pressure was estimated by measuring the diameter of the inferior vena cava and its collapsibility during inspiration. Systolic pulmonary arterial pressure (sPAP) was calculated by adding the estimated right atrial pressure to the maximum systolic pressure gradient derived from tricuspid regurgitation velocity. The left atrial volume index was calculated using Simpson’s method from apical 4-chamber and 2-chamber views. Mitral, aortic, and tricuspid valve regurgitations were assessed by measuring the vena contracta in the apical 4-chamber view.

### 4.3. Exercise Echocardiography

Exercise stress echocardiography (ESE) was performed by an experienced cardiologist, on a semirecumbent cycle ergometer (Ergoline, model 900 EL, Saarbrücken, Germany) with an incremental workload of 25 W every 2 min up to the symptom-limited maximal tolerated workload [22]. In subjects with reduced functional capacity, the exercise protocol allowed for lower incremental workloads (10–20 WU every 2 min), as specified in the records. Key echocardiographic measurements were acquired at baseline and peak exercise, including but not limited to RV function (TAPSE, sPAP, CO, and pulmonary vascular resistance [PVR]). Cardiac output (CO) was calculated as heart rate (HR) × stroke volume, the latter obtained through Doppler analysis of the left ventricular outflow tract. mPAP was calculated as 0.6 × sPAP + 2 mm Hg [23]. PVR was calculated as mPAP minus wedged PAP estimated from the trans-mitral E Doppler flow to mitral annulus tissue Doppler e’ ratio divided by CO. During the exercise, heart rate by electrocardiogram and blood pressure by sphygmomanometer were continuously monitored at baseline and during the last 15 s of each workload step. Termination criteria and/or positive test criteria for inducible myocardial ischemia conformed to current recommendations [24]. Exercise PH was defined as a steep increase in mean pulmonary arterial pressure (mPAP) with a mPAP/cardiac output (CO) slope > 3 mmHg/min/L. Inter- and intra-observer variability for echocardiographic measurements was not specifically investigated.

### 4.4. Cardiopulmonary Exercise Testing

Cardiopulmonary exercise testing (CPET) was performed using an electronically braked cycle ergometer and the Vmax 6200 Spectra Series software (SensorMedics, Hochberg, Germany, https://www.bioclinicalservices.com.au/sensormedics, accessed on 19 October 2025), following a progressively increasing workload protocol. The test was interrupted when any of the following symptoms or signs occurred: angina; electrocardiographic signs of myocardial ischemia or injury; excessive blood pressure increase (systolic blood pressure ≥ 240 mmHg, diastolic blood pressure ≥ 120 mmHg); dyspnea; or achievement of the maximal predicted heart rate. ExPH during CPET was defined as a combination of abnormal maximal oxygen consumption (VO_2_ max), reduced peak O_2_ pulse, and abnormal changes between rest and exercise in the following parameters: minute ventilation/carbon dioxide production (Ve/VCO_2_ slope), minute ventilation/oxygen consumption (Ve/VO_2_ slope), peak heart rate/oxygen consumption (HR/VO_2_ slope), dead space-to-tidal volume ratio (VD/VT), and end-expiratory CO_2_ pressure (PETCO_2_).

### 4.5. Statistical Analysis

Categorical data were described with the absolute frequency, and continuous data were summarized with the mean and standard deviation. To compare qualitative and quantitative parameters with a Q-scan (positive, negative), a chi-square test and the *t*-test for independent samples (two-tailed) were used, respectively. Temporal changes in continuous echocardiographic parameters between the two groups were assessed using repeated-measures ANOVA. The level of statistical significance was set at 0.05, and all statistical analyses were performed using IBM^®^ SPSS^®^ software, version 29.

## 5. Conclusions

The persistence of perfusion defects on a lung Q-scan after acute PE is associated with subclinical right ventricular dysfunction and altered pulmonary hemodynamics, detectable through echocardiography and stress testing despite normal resting findings. The non-invasive detection of exercise-induced pulmonary hypertension (ExPH) via CPET and ESE correlates with Q-scan positivity and helps identify a high-risk PPES phenotype. These results support the use of multimodal, long-term follow-up to detect early signs of disease progression toward CTEPD with/without PH and refine post-PE risk stratification.

## Figures and Tables

**Table 1:** Baseline clinical characteristics of the study population stratified by lung Q-scan.

Clinical Features	Q-Scan Negative (Mean ± SD or n/%)	Q-Scan Positive (Mean ± SD or n/%)	*p*-Value
**Sex**			0.064
Female	8	23	
Male	12	12	
**Age**	59.40 (10.80)	67.26 (18.68)	0.053
**BMI**	26.76 (4.69)	26.86 (5.01)	0.94
**Smoking history**			0.839
None or quit > 20 years	10	18	
Quit < 20 years	3	7	
Yes	7	10	
**SAH**			0.325
Absent	18	28	
Present	2	7	
**Dyslipidemia**			0.234
Absent	18	27	
Present	2	8	
**Diabetes Mellitus**			0.624
Absent	19	32	
Present	1	3	
**Vascular events**			**0.009**
Absent	11	7	
SVT + DVT	0	11	
PE	3	8	
DVT + PE	6	9	
**Thyroid disease**			0.276
Absent	20	33	
Hypothyroidism	0	2	
**COPD**			0.475
Absent	19	31	
Present	1	4	
**Oncologic history**			0.082
No	17	22	
Yes	3	13	
**Known thrombophilia**			0.057
No	18	35	
Yes	2	0	
**Hematologic diseases**			0.446
Absent	20	34	
Notspecified	0	1	
**Family history of PAH**			0.276
No	20	33	
Yes	0	2	
**AoCo bypass**			0.446
No	20	34	
Yes	0	1	
**Valve surgery**			0.276
No	20	33	
Yes	0	2	
**Single-vessel CAD**			0.182
No	19	35	
Yes	1	0	
**Three-vessel CAD**			0.446
No	20	34	
Yes	0	1	
**Family history of CAD**			0.556
No	18	33	
Yes	2	2	
**Beta-blockers**			0.335
No	18	28	
Yes	2	7	
**Antiarrhythmics**			0.446
No	20	34	
Yes	0	1	
**Sartans**			0.425
No	19	31	
Yes	1	4	
**ACE inhibitors**			0.425
No	19	31	
Yes	1	4	
**Calcium channel blockers**			0.276
No	20	33	
Yes	0	2	
**Diuretics**			0.556
No	18	33	
Yes	2	2	
**Lipid-lowering drugs**			0.425
No	19	31	
Yes	1	4	
**Antidiabetic drugs**			0.425
No	19	31	
Yes	1	4	
**Temporary PE risk factors**			**0.022**
No	19	24	
Yes	1	11	
**Permanent PE risk factors**			0.356
No	15	22	
Yes	5	13	
**Unprovoked PE**			**0.012**
No	5	21	
Yes	15	14	
**Estroprogestin use**			0.036
No	19	25	
Yes	1	10	

Abbreviations: BMI, body mass index; SAH, systemic arterial hypertension; SVT, superficial venous thrombosis; DVT, deep venous thrombosis; PE, pulmonary embolism; COPD, chronic obstructive pulmonary disease; PAH, pulmonary arterial hypertension; CAD, coronary artery disease.

**Table 2:** Clinical characteristics at the time of the acute event stratified by lung Q-scan.

Clinical Features	Q-Scan Negative (Mean ± SD or n/%)	Q-Scan Positive (Mean ± SD or n/%)	*p*-Value
**HR (bpm)**	67.05 (10.25)	91.06 (22.43)	**<0.001**
**SBP (mmHg)**	118.45 (13.57)	110.26 (17.86)	0.062
**DBP (mmHg)**	76.10 (6.21)	67.74 (14.92)	**0.006**
**NT-proBNP**	633.55 (2309.90)	5187.31 (8088.77)	**0.003**
**WHO functional class**			**<0.001**
1	12	6	
2	5	5	
3	2	24	
4	1	0	
**Heart failure**			**0.006**
No	14	11	
Yes	6	24	
**Syncope**			0.047
No	18	23	
Yes	2	12	
**Resting PH probability**			**<0.001**
Low	17	0	
Intermediate	3	1	
High	0	34	
**PESI score at admission**			**<0.001**
Low	16	9	
Intermediate	2	7	
High	2	19	
**Fibrinolysis**			**0.001**
No	20	21	
Yes	0	14	
**NOAC**			0.239
No	7	18	
Yes	13	17	
**IVC filter**			0.182
No	19	35	
Yes	1	0	
**Heparin**			**0.003**
No	19	20	
Yes	1	15	
**AngioCT acute phase**			**<0.001**
Mild	14	6	
Submassive	4	6	
Massive	2	23	
**Tricuspid regurgitation**			0.262
No	4	12	
Yes	16	23	
**Mitral regurgitation**			0.714
No	7	14	
Yes	13	21	
**Aortic regurgitation**			0.116
No	20	31	
Yes	0	4	
**Aortic stenosis**			0.446
No	20	34	
Yes	0	1	

Abbreviations: HR, heart rate (bpm); SBP; systolic arterial pressure (mmHg); DBP, diastolic arterial pressure (mmHg); PESI, pulmonary embolism severity index; NOAC, novel oral anticoagulants; IVC filter, inferior venous cava filter.

**Table 3:** Transthoracic echocardiographic findings at hospital discharge after acute pulmonary embolism stratified by lung Q-scan.

TTE Findings	Q-Scan Negative (Mean ± SD or n/%)	Q-Scan Positive (Mean ± SD or n/%)	*p*-Value
**LVEDD**	46.50 (7.56)	50.11 (6.58)	0.069
**LVESD**	27.61 (8.95)	29.94 (6.64)	0.326
**LVEDV**	117.05 (13.93)	119.49 (30.20)	0.685
**LVESV**	50.65 (18.48)	41.80 (16.16)	0.069
**LV mass**	135.15 (15.15)	156.51 (28.57)	**0.003**
**LAD**	40.40 (9.49)	43.29 (4.81)	0.216
**LAV**	25.55 (11.54)	46.83 (10.42)	**<0.001**
**LVEF**	55.95 (13.26)	65.00 (8.19)	**0.010**
**FwSV LVOT**	80.95 (10.32)	69.17 (7.61)	**0.009**
**MR**	1.25 (0.85)	1.06 (0.94)	0.452
**AO disease**	0.25 (0.44)	0.77 (0.91)	**0.006**
**LV E/A**	1.18 (0.46)	0.98 (0.44)	0.083
**LV E/e’**	14.25 (1.25)	6.63 (2.18)	**<0.001**
**RD1**	35.15 (7.56)	53.31 (3.64)	**<0.001**
**RD2**	29.30 (7.96)	51.26 (5.38)	**<0.001**
**RD3**	21.35 (4.90)	49.37 (6.02)	**<0.001**
**RVOTprox**	24.70 (5.87)	44.37 (4.87)	**<0.001**
**RVOTdist**	23.65 (5.46)	43.31 (5.60)	**<0.001**
**Eccentricity index**	1.05 (0.12)	0.67 (0.16)	**<0.001**
**RV/LV diameter ratio**	0.89 (0.11)	0.77 (0.13)	**<0.001**
**TAPSE**	22.10 (4.85)	14.29 (2.28)	**<0.001**
**FAC**	50.70 (10.63)	31.34 (5.65)	**<0.001**
**RV E/A**	1.41 (0.26)	0.63 (0.18)	**<0.001**
**RV E/e’**	8.20 (1.70)	16.77 (1.14)	**<0.001**
**TR**	1.60 (0.60)	1.26 (0.74)	0.084
**sPAP**	27.45 (10.11)	44.74 (2.60)	**<0.001**
**mPAP**	18.65 (6.27)	29.00 (1.64)	**<0.001**
**TR velocity**	2.01 (0.49)	3.49 (0.27)	**<0.001**
**RVOT AT**	114.55 (12.52)	61.31 (10.61)	**<0.001**
**IVC diameter**	16.30 (1.08)	24.49 (3.17)	**<0.001**
**Right atrial area**	15.50 (2.67)	16.31 (2.32)	0.241
**TAPSE/sPAP**	0.98 (0.42)	0.32 (0.06)	**<0.001**
**IVC collapsibility**			0.178
No	0	3	
Yes	20	32	

Abbreviations: LVEDD, left ventricular end diastolic diameter; LVESD, left ventricular end systolic diameter; LVEDV, left ventricular end diastolic volume; LVESV, left ventricular end systolic volume; LAD, left atrial diameter; LAV, left atrial volume; LVEF, left ventricular ejection fraction; FwSV, forward stroke volume; LVOT, left ventricular outflow tract; MR, mitral regurgitation; AO, aorta; A wave, peak filling flow velocity of the ventricle in late diastole (atrial contraction); E wave, peak filling flow velocity of the ventricle in early diastole (passive filling); e’ wave, early diastolic velocity of the mitral or tricuspid annulus; RD1, right ventricular basal diameter; RD2 right mid-ventricular diameter; RD3, right ventricular longitudinal diameter; RVOT, right ventricular outflow tract; TAPSE, tricuspid annular plane systolic excursion; FAC, fractional area change; TR, tricuspid regurgitation; sPAP, systolic pulmonary arterial pressure; mPAP, mean pulmonary arterial pressure; TRV tricuspid regurgitation velocity; RVOT AT, right ventricular outflow tract acceleration time; IVC, inferior vena cava.

**Table 4:** Clinical, functional, and hemodynamic parameters at 4-month follow-up stratified by lung Q-scan.

Clinical Features	Q-Scan Negative (Mean ± SD or n/%)	Q-Scan Positive (Mean ± SD or n/%)	*p*-Value
**WHO Functional Class**			**0.013**
1	10	5	
2	6	14	
3	4	16	
**Heart failure**			0.194
No	19	29	
Yes	1	6	
**CPET compatible with ex-PH**			**<0.001**
No	17	4	
Yes	0	24	
**Resting PH probability**			**<0.001**
1	19	0	
2	1	35	
**ESE compatible with ex-PH**			**<0.001**
No	16	0	
Yes	0	24	
**HR (bpm)**	76.2 (14.7)	72.7 (13.2)	0.377
**SBP (mmHg)**	126.6 (14.2)	128.0 (16.7)	0.747
**DBP (mmHg)**	75.7 (7.3)	75.4 (9.6)	0.919
**NT-proBNP**	94.7 (134.4)	1256.2 (562.5)	**<0.001**
**N. seg defect by P scan**	0.5 (0.9)	2.9 (1.1)	**<0.001**
**6MWT distance**	684.6 (52.1)	469.2 (146.6)	**<0.001**

Abbreviations: WHO, World Health Organization; Ex-PH, exercise-induced pulmonary hypertension; CPET, cardiopulmonary exercise test; HR, heart rate (bpm); SBP; systolic arterial pressure (mmHg); DBP, diastolic arterial pressure (mmHg); NT-proBNP, N-terminal pro natriuretic peptide; 6MWT, six-minute walking test.

**Table 5:** Comparison of the CPET findings at 4 months after the acute pulmonary embolism event stratified by lung Q-scan.

CPET Findings	Q-Scan Negative (Mean ± SD or n/%)	Q-Scan Positive (Mean ± SD or n/%)	*p*-Value
**CPET compatible with ex-PH**			**<0.001**
No	17	4	
Yes	0	24	
**Peak VO_2_**	23.25 (3.98)	15.75 (4.27)	**<0.001**
**VE/VCO_2_ slope**	18.00 (4.19)	34.00 (6.52)	**<0.001**
**Peak O_2_ pulse**	23.62 (5.38)	8.81 (3.49)	**<0.001**
**VD/VT**	0.87 (0.64)	0.24 (0.12)	**<0.001**
**HR/VO_2_ slope**	2.90 (1.73)	6.72 (2.81)	**<0.001**
**PetCO_2_**	6.39 (8.94)	6.21 (3.40)	0.921

Abbreviations: Ex-PH, exercise-induced pulmonary hypertension; CPET, cardiopulmonary exercise test; Peak VO_2_, peak oxygen consumption; VE/VCO_2_ slope, minute ventilation/carbon dioxide production difference between rest and stress; VD/VT, dead space over tidal volume; HR, heart rate; PetVCO_2_, capnography.

**Table 6:** Transthoracic echocardiographic findings at 4 months after the acute pulmonary embolism event stratified by lung Q-scan.

TTE Findings	Q-Scan Negative (Mean ± SD or n/%)	Q-Scan Positive (Mean ± SD or n/%)	*p*-Value
**LVEDD**	46.60 (8.17)	52.20 (7.21)	0.011
**LVESD**	28.75 (7.45)	32.23 (7.14)	0.093
**LVEDV**	117.65 (20.26)	120.94 (27.32)	0.641
**LVESV**	47.50 (10.90)	42.89 (15.38)	0.243
**LV mass**	136.05 (14.71)	158.00 (26.05)	**0.001**
**LAD**	38.35 (11.17)	44.26 (4.83)	0.034
**LAV**	40.40 (9.21)	47.14 (9.28)	**0.012**
**LVEF**	59.30 (6.68)	64.14 (7.65)	**0.022**
**FwSV LVOT**	72.87 (10.27)	66.46 (5.69)	**0.036**
**MR**	1.25 (0.91)	1.09 (0.92)	0.525
**AO disease**	0.25 (0.55)	0.80 (0.90)	**0.007**
**LV E/A**	1.04 (0.40)	0.92 (0.38)	0.269
**LV E/e’**	11.30 (2.25)	7.31 (1.98)	**<0.001**
**RD1**	30.45 (7.04)	46.63 (5.13)	**<0.001**
**RD2**	27.55 (6.12)	44.77 (6.02)	**<0.001**
**RD3**	22.80 (4.61)	42.77 (6.32)	**<0.001**
**RVOT prox**	26.30 (2.87)	39.26 (5.54)	**<0.001**
**RVOT dist**	25.70 (3.66)	38.97 (5.99)	**<0.001**
**Eccentricity index**	1.14 (0.09)	0.65 (0.13)	**<0.001**
**RV/LV diameter ratio**	0.85 (0.10)	0.71 (0.12)	**<0.001**
**TAPSE**	22.00 (3.26)	15.29 (2.01)	**<0.001**
**FAC**	56.60 (10.56)	32.94 (5.26)	**<0.001**
**RV E/A**	1.61 (0.25)	0.65 (0.14)	**<0.001**
**RV E/e’**	8.10 (1.83)	17.23 (1.24)	**<0.001**
**TR**	1.45 (0.69)	1.23 (0.73)	0.274
**sPAP**	24.10 (6.24)	43.29 (2.18)	**<0.001**
**mPAP**	16.12 (4.30)	28.00 (2.14)	**<0.001**
**TR velocity**	1.94 (0.45)	3.32 (0.10)	**<0.001**
**RVOT AT**	118.00 (12.49)	65.86 (9.54)	**<0.001**
**VCI diameter**	17.25 (0.72)	19.71 (4.06)	**0.001**
**Right atrial area**	15.90 (2.20)	16.89 (1.76)	0.074
**TAPSE/sPAP**	1.01 (0.42)	0.35 (0.05)	**<0.001**
**IVC collapsibility**			0.116
No	20	31	
Yes			
**ΔmPAP/CO**	2.09 (0.36)	6.40 (1.28)	**<0.001**

Abbreviations: LVEDD, left ventricular end diastolic diameter; LVESD, left ventricular end systolic diameter; LVEDV, left ventricular end diastolic volume; LVESV, left ventricular end systolic volume; LAD, left atrial diameter; LAV, left atrial volume; LVEF, left ventricular ejection fraction; FwSV, forward stroke volume; LVOT, left ventricular outflow tract; MR, mitral regurgitation; AO, aorta; A wave, peak filling flow velocity of the ventricle in late diastole (atrial contraction); E wave, peak filling flow velocity of the ventricle in early diastole (passive filling); e’ wave, early diastolic velocity of the mitral or tricuspid annulus; RD1, right ventricular basal diameter; RD2 right mid-ventricular diameter; RD3, right ventricular longitudinal diameter; RVOT, right ventricular outflow tract; TAPSE, tricuspid annular plane systolic excursion; FAC, fractional area change; TR, tricuspid regurgitation; sPAP, systolic pulmonary arterial pressure; mPAP, mean pulmonary arterial pressure; TRV tricuspid regurgitation velocity; RVOT AT, right ventricular outflow tract acceleration time; IVC, inferior vena cava; ΔmPAP/CO slope, delta between rest and stress of the pulmonary artery mean pressure over cardiac output ratio.

**Table 7:** Clinical, functional, and hemodynamic parameters at 48-month follow-up stratified by lung Q-scan.

Clinical Features	Q-Scan Negative (Mean ± SD or n/%)	Q-Scan Positive (Mean ± SD or n/%)	*p*-Value
**WHO Functional Class**			**<0.001**
1	18	7	
2	2	12	
3	0	16	
**Heart failure**			0.178
No	20	32	
Yes	0	3	
**Resting PH probability**			**<0.001**
1	19	9	
2	1	25	
3	0	1	
**HR (bpm)**	74.15 (7.95)	76.29 (8.29)	0.355
**SBP (mmHg)**	131.20 (8.28)	134.63 (9.59)	0.186
**DBP (mmHg)**	73.15 (4.25)	77.91 (5.69)	**<0.001**

Abbreviations: WHO, World Health Organization; PH, pulmonary hypertension; HR, heart rate (bpm); SBP; systolic arterial pressure (mmHg); DBP, diastolic arterial pressure (mmHg).

**Table 8:** Transthoracic echocardiographic findings at 48 months after the acute pulmonary embolism event stratified by lung Q-scan.

TTE Findings	Q-Scan Negative (Mean ± SD or n/%)	Q-Scan Positive (Mean ± SD or n/%)	*p*-Value
**LVEDD**	46.10 (6.73)	47.40 (8.42)	0.557
**LVESD**	28.80 (6.06)	30.91 (6.67)	0.248
**LVEDV**	111.00 (24.03)	124.54 (27.65)	0.073
**LVESV**	43.25 (12.96)	43.20 (15.85)	0.990
**LV mass**	162.45 (47.74)	157.46 (22.40)	0.663
**LAD**	39.50 (9.07)	42.31 (5.75)	0.164
**LAV**	39.00 (7.69)	47.94 (9.64)	**0.001**
**LVEF**	60.40 (8.98)	64.74 (9.96)	0.113
**FwSV LVOT**	67.50 (11.94)	69.00 (6.89)	0.611
**MR**	1.25 (0.85)	1.11 (0.93)	0.594
**AO disease**	0.20 (0.52)	0.80 (0.96)	**0.004**
**E wave**	0.73 (0.31)	0.65 (0.12)	0.309
**LV E/e′**	13.80 (1.54)	7.10 (2.24)	**<0.001**
**RD1**	31.30 (5.22)	39.43 (11.82)	**0.001**
**RD2**	29.25 (4.62)	36.31 (10.77)	**0.001**
**RD3**	27.10 (4.47)	32.80 (11.50)	**0.012**
**RVOT prox**	23.95 (4.25)	32.89 (8.49)	**<0.001**
**RVOT dist**	23.00 (4.22)	33.06 (8.26)	**<0.001**
**Eccentricity index**	1.13 (0.08)	1.03 (0.29)	0.078
**RV/LV diameter ratio**	0.85 (0.08)	0.78 (0.13)	**0.016**
**TAPSE**	23.50 (3.07)	19.57 (3.42)	**<0.001**
**FAC**	59.40 (5.09)	37.40 (8.98)	**<0.001**
**RV E/A**	1.56 (0.24)	0.72 (0.23)	**<0.001**
**RV E/e’**	6.90 (1.74)	24.86 (2.64)	**<0.001**
**TR**	1.35 (0.75)	1.20 (0.76)	0.481
**sPAP**	21.05 (4.62)	39.46 (7.07)	**<0.001**
**mPAP**	14.75 (2.81)	25.89 (4.39)	**<0.001**
**TR velocity**	2.23 (0.39)	2.92 (0.40)	**<0.001**
**RVOT AT**	118.75 (9.48)	85.71 (25.45)	**<0.001**
**IVC diameter**	16.40 (1.47)	21.14 (3.19)	**<0.001**
**Right atrial area**	15.67 (2.09)	21.66 (3.52)	**<0.001**
**TAPSE/sPAP**	1.15 (0.31)	0.53 (0.18)	**<0001**
**IVC collapsibility**			0.276
No	0	2	
Yes	20	33	

Abbreviations: LVEDD, left ventricular end diastolic diameter; LVESD, left ventricular end systolic diameter; LVEDV, left ventricular end diastolic volume; LVESV, left ventricular end systolic volume; LAD, left atrial diameter; LAV, left atrial volume; LVEF, left ventricular ejection fraction; FwSV, forward stroke volume; LVOT, left ventricular outflow tract; MR, mitral regurgitation; AO, aorta; A wave, peak filling flow velocity of the ventricle in late diastole (atrial contraction); E wave, peak filling flow velocity of the ventricle in early diastole (passive filling); e’ wave, early diastolic velocity of the mitral or tricuspid annulus; RD1, right ventricular basal diameter; RD2 right mid-ventricular diameter; RD3, right ventricular longitudinal diameter; RVOT, right ventricular outflow tract; TAPSE, tricuspid annular plane systolic excursion; FAC, fractional area change; TR, tricuspid regurgitation; sPAP, systolic pulmonary arterial pressure; mPAP, mean pulmonary arterial pressure; TRV, tricuspid regurgitation velocity; RVOT AT, right ventricular outflow tract acceleration time; IVC, inferior vena cava.

**Table 9:** Long-term variation (4 to 48 months) in echocardiographic parameters stratified by lung Q-scan.

ΔTTE Findings (t1–t4)	Q-Scan Negative (Mean ± SD or n/%)	Q-Scan Positive (Mean ± SD or n/%)	*p*-Value
**WHO Functional Class**	−20.83 (40.78)%	8.57 (49.90)%	**0.029**
**LVEF**	2.69 (17.06)%	1.36 (12.71)%	0.074
**RD1**	4.79 (13.02) mm	−15.86 (22.03) mm	**<0.001**
**RD2**	7.95 (13.66) mm	−18.57 (23.46) mm	**<0.001**
**RD3**	21.69 (22.60) mm	−22.85 (26.01) mm	**<0.001**
**RVOT prox**	−8.59 (14.33) mm	−16.51 (15.83) mm	0.071
**RVOT dist**	−10.13 (14.41) mm	−14.78 (17.73) mm	0.296
**Eccentricity index**	−0.69 (4.57)	65.38 (62.47)	**<0.001**
**RV/LV diameter ratio**	0.46 (5.76)	11.66 (20.85)	**0.005**
**TAPSE**	9.03 (22.81) mm	30.46 (29.73) mm	**0.007**
**FAC**	8.25 (21.26)	13.89 (21.71)	0.354
**RV E/A**	13.74 (36.72)	−2.14 (12.88)	**0.025**
**RV E/e’**	−13.11 (18.97)	45.04 (19.26)	**<0.001**
**TR**	−5.56 (23.57) mL	−6.90 (34.65) mL	0.886
**sPAP**	−7.46 (26.03) mmHg	−8.79 (16.09) mmHg	0.838
**mPAP**	0.79 (47.20) mmHg	−7.17 (16.08) mmHg	0.473
**TR velocity**	17.75 (17.34) m/s	−12.07 (11.86) m/S	**<0.001**
**RVOT AT**	1.45 (11.10) s	29.76 (33.32) s	**<0.001**
**IVC diameter**	−4.78 (9.21) mm	10.45 (22.64) mm	**0.001**
**Right atrial area**	2.33 (17.38) mm^2^	29.20 (22.95) mm^2^	**<0.001**
**TAPSE/sPAP**	28.72 (63.69) mm/mmHg	55.45 (61.82) mm/mmHg	0.133

Abbreviations: WHO, World Health Organization; PH, pulmonary hypertension; LVEF, left ventricular ejection fraction; RD1, right ventricular basal diameter; RD2, right mid-ventricular diameter; RD3, right ventricular longitudinal diameter; RVOT, right ventricular outflow tract; TAPSE, tricuspid annular plane systolic excursion; FAC, fractional area change; A wave, peak filling flow velocity of the ventricle in late diastole (atrial contraction); E wave, peak filling flow velocity of the ventricle in early diastole (passive filling); e’ wave, early diastolic velocity of the mitral or tricuspid annulus; TR, tricuspid regurgitation; sPAP, systolic pulmonary arterial pressure; mPAP, mean pulmonary arterial pressure; TRV, tricuspid regurgitation velocity; RVOT AT, right ventricular outflow tract acceleration time; IVC, inferior vena cava.

## Data Availability

The original contributions presented in this study are included in the article/supplementary material. Further inquiries can be directed to the corresponding authors.

## Supplementary Materials

The following supporting information can be downloaded at: https://www.mdpi.com/article/10.3390/ijms262110617/s1.

## Abbreviations

CPET	cardiopulmonary exercise test
CTEPD	chronic thromboembolic pulmonary disease
CTEPH	chronic thromboembolic pulmonary hypertension
ESE	exercise stress echocardiography
Ex-PH	exercise-induced pulmonary hypertension
mPAP	mean pulmonary arterial pressure
PAPs	systolic pulmonary artery pressure
PE	pulmonary thromboembolism
PPES	post-PE syndrome
Q-scan	perfusion scan
RHC	right heart catheterization
RV	right ventricle
TTE	transthoracic echocardiography
