# Supplementary material for: Chronic Thromboembolic Pulmonary Disease: Right Ventricular Function and Pulmonary Hemodynamics in a 4-Year Follow-Up
Source: Int J Mol Sci. 2025 Oct 31;26(21):10617. doi: 10.3390/ijms262110617 (PMC12610875; doi:10.3390/ijms262110617)
Supplement: Supplementary file 1 [file ijms-26-10617-s001.zip › ijms-3870364-supplementary.pdf]

Online supplement text

Materials and methods

Study design and data collection

This was a prospective, observational, cohort, single-center study that enrolled patients with an history of pulmonary embolism (PE) at our Cardiology Division. The study was conducted in accordance with the Helsinki Declaration, and all patients provided informed consent before each diagnostic test, which was carried out exclusively for clinical purposes. Local investigators had full access to patient data and medical records. Enrollment took place between 2020 and 2024, and 55 patients were included based on the following inclusion criteria: diagnosis of acute pulmonary embolism, eligibility for effective post-PE anticoagulation, age between 18 and 85 years. Patients with the following criteria were excluded: conditions that prevent the performance of exercise testing, limited life expectancy, moderate to severe primary tricuspid regurgitation. Patients were selected at different time points among those admitted to the cardiology Division of the University Hospital of Cisanello, Pisa University, in Pisa Italy, with a diagnosis of PE in 2020. At discharge, patients were prescribed anticoagulation therapy in accordance with current guidelines for PE. After 4–6 months, patients who had completed an effective anticoagulation course, were screened for symptoms of cardiorespiratory impairment attributable to the index PE event and underwent lung perfusion scintigraphy (Q-scan). Instead of ventilation scintigraphy, a chest X-ray was performed as per routine practice in our Nuclear Medicine Unit. This procedure is permitted in clinical practice by the 2015 ESC guidelines and remains in effect with the 2022 guidelines in centers that cannot rely on V/Q scintigraphy, being supported by solid evidence [10,21]. Patients were classified as Q-scan-negative (group 1, n=20) or Q-scan-positive (group 2, n=35). Both groups underwent transthoracic echocardiography (TTE) at rest, and were subclassified based on the probability of pulmonary hypertension (PH) as high, intermediate, or low. Patients with high PH probability (n=3) were excluded and informed of the need for right heart catheterization (RHC). Those with intermediate or low PH probability underwent exercise testing, with exercise echocardiography (ESE) performed on the same day as TTE. CPET was carried out in a second session, almost two weeks after the acute event, to optimize patient compliance and allow for adequate physical recovery. In accordance with current PH guidelines (ESC/ERS 2015 and 2019), patients with a positive Q-scan, intermediate PH

probability, and positive CPET were informed about the possibility of undergoing RHC and instructed on the risk-benefit ratio of the test. Group 1 and group 2 were compared based on the following parameters:

1. Extent of thrombotic load of PE on Q-scan (number of segments with perfusion defect) and contrast-enhanced CT pulmonary angiography (mild, sub-massive, or massive PE according to the American Heart Association definitions [11];
2. Presence of thrombophilia (limited to tests not affected by anticoagulation, namely factor V Leiden, prothrombin variant, anti-phospholipid, and anti-beta-2-glycoprotein antibodies);
3. Cardiovascular risk factors;
4. Anthropometric and demographic parameters;
5. Anticoagulation treatment in the hospital and at discharge.

The two patient groups were also compared for World Health Organization functional class (WHO-FC), NT-proBNP, CPET parameters, and echocardiographic parameters at rest and at exercise (ESE).

Subsequently, enrolled patients were followed up at 24, 36, and 48 months post-admission, during which a resting TTE, clinical examination, and blood tests were repeated.

Resting Echocardiography

Mono- and two-dimensional transthoracic echocardiography (TTE) was performed using a Philips iE33 echocardiograph (Philips xMATRIX echocardiography system, Andover, MA, USA). Images were recorded over at least 3 cardiac cycles. The echocardiographic probability of pulmonary hypertension (PH) was assessed in according to current guideline criteria [1]. Right atrial pressure was estimated by measuring the diameter of the inferior vena cava and its collapsibility during inspiration. Systolic pulmonary arterial pressure (sPAP) was calculated by adding the estimated right atrial pressure to the maximum systolic pressure gradient derived from tricuspid regurgitation velocity. The left atrial volume index was calculated using Simpson’s method from apical 4-chamber and 2-chamber views. Mitral, aortic, and tricuspid valve regurgitations were assessed by measuring the vena contracta in the apical 4-chamber view.

Exercise Echocardiography

Exercise stress echocardiography (ESE) was performed on a semirecumbent cycle ergometer (Ergoline, model 900 EL, Saarbrücken, Germany). with an incremental

workload of 25 W every 2 minutes up to the symptom-limited maximal tolerated workload [22]. In subjects with reduced functional capacity, the exercise protocol allowed for lower incremental workloads (10-20 WU every 2 minutes), as specified in the records. Key echocardiographic measurements were acquired at baseline and peak exercise, including but not limited to RV function (TAPSE, sPAP, CO, and pulmonary vascular resistance [PVR]). Cardiac output (CO) was calculated as heart rate (HR) × stroke volume, the latter obtained through Doppler analysis of the left ventricular outflow tract. mPAP was calculated as: 0.6 × sPAP +2 mm Hg [23]. PVR was calculated as mPAP minus wedged PAP estimated from the trans-mitral E Doppler flow to mitral annulus tissue Doppler e' ratio divided by CO. During the exercise, heart rate by electrocardiogram and blood pressure by sphygmomanometer were continuously monitored at baseline and during the last 15 seconds of each workload step. Termination criteria and/or positive test criteria for inducible myocardial ischemia conformed to current recommendations [24]. Exercise PH was defined as steep increase in mean pulmonary arterial pressure (mPAP) with a mPAP/cardiac output (CO) slope >3 mmHg.min/L.

Cardiopulmonary Exercise Testing

Cardiopulmonary exercise testing (CPET) was performed using an electronically braked cycle ergometer and the Vmax 6200 Spectra Series software (SensorMedics, Hochberg, Germany), following a progressively increasing workload protocol. The test was interrupted when any of the following symptoms or signs occurred: angina; electrocardiographic signs of myocardial ischemia or injury; excessive blood pressure increase (systolic blood pressure ≥ 240 mmHg, diastolic blood pressure ≥ 120 mmHg); dyspnea; or achievement of the maximal predicted heart rate. ExPH during CPET was defined as a combination of abnormal maximal oxygen consumption (VO_2 max), reduced peak O_2 pulse, and abnormal changes between rest and exercise in the following parameters: minute ventilation/carbon dioxide production (Ve/VCO_2 slope), minute ventilation/oxygen consumption (Ve/VO_2 slope), peak heart rate/oxygen consumption (HR/VO_2 slope), dead space to tidal volume ratio (VD/VT), and end-expiratory CO_2 pressure ($PETCO_2$).
